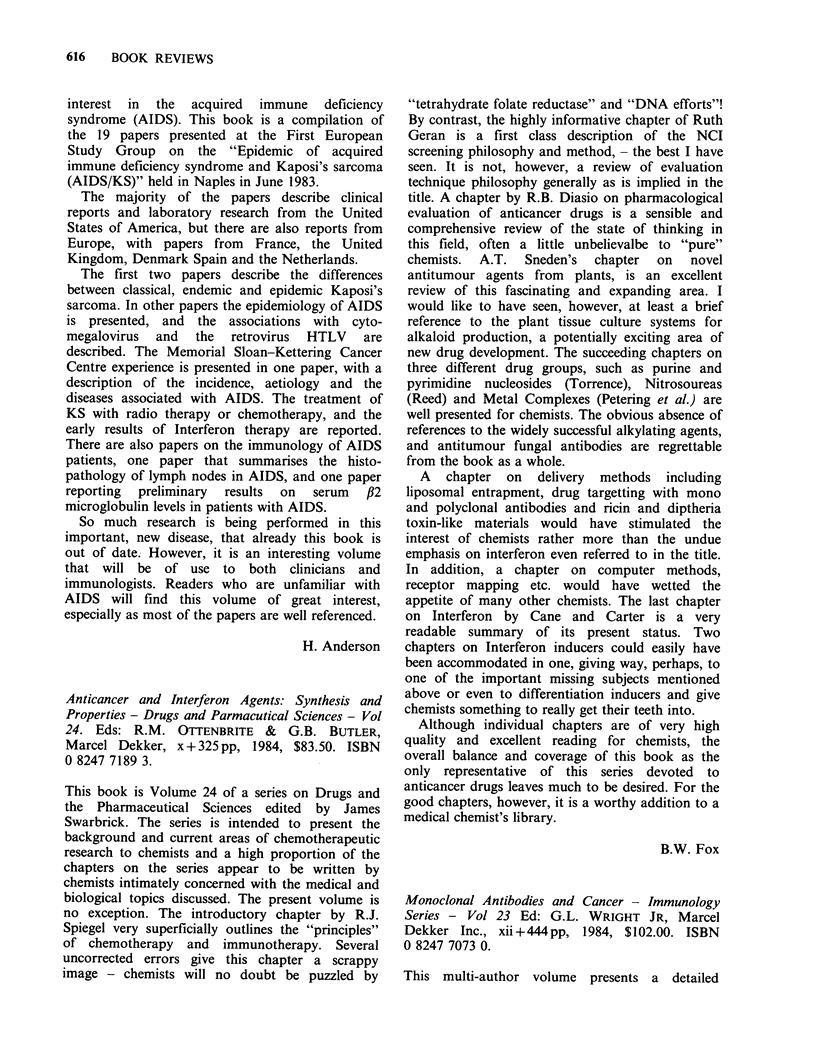# Anticancer and Interferon Agents: Synthesis and Properties - Drugs and Parmacutical Sciences - Vol. 24

**Published:** 1985-04

**Authors:** B.W. Fox


*Anticancer and Interferon Agents: Synthesis and Properties – Drugs and Parmacutical Sciences – Vol 24.* Eds: R.M. Ottenbrite & G.B. Butler, Marcel Dekker, x+325 pp, 1984, $83.50. ISBN 0 8247 7189 3.

This book is Volume 24 of a series on Drugs and the Pharmaceutical Sciences edited by James Swarbrick. The series is intended to present the background and current areas of chemotherapeutic research to chemists and a high proportion of the chapters on the series appear to be written by chemists intimately concerned with the medical and biological topics discussed. The present volume is no exception. The introductory chapter by R.J. Spiegel very superficially outlines the "principles" of chemotherapy and immunotherapy. Several uncorrected errors give this chapter a scrappy image – chemists will no doubt be puzzled by "tetrahydrate folate reductase" and "DNA efforts"! By contrast, the highly informative chapter of Ruth Geran is a first class description of the NCI screening philosophy and method, – the best I have seen. It is not, however, a review of evaluation technique philosophy generally as is implied in the title. A chapter by R.B. Diasio on pharmacological evaluation of anticancer drugs is a sensible and comprehensive review of the state of thinking in this field, often a little unbelievalbe to "pure" chemists. A.T. Sneden's chapter on novel antitumour agents from plants, is an excellent review of this fascinating and expanding area. I would like to have seen, however, at least a brief reference to the plant tissue culture systems for alkaloid production, a potentially exciting area of new drug development. The succeeding chapters on three different drug groups, such as purine and pyrimidine nucleosides (Torrence), Nitrosoureas (Reed) and Metal Complexes (Petering *et al.)* are well presented for chemists. The obvious absence of references to the widely successful alkylating agents, and antitumour fungal antibodies are regrettable from the book as a whole.

A chapter on delivery methods including liposomal entrapment, drug targetting with mono and polyclonal antibodies and ricin and diptheria toxin-like materials would have stimulated the interest of chemists rather more than the undue emphasis on interferon even referred to in the title. In addition, a chapter on computer methods, receptor mapping etc. would have wetted the appetite of many other chemists. The last chapter on Interferon by Cane and Carter is a very readable summary of its present status. Two chapters on Interferon inducers could easily have been accommodated in one, giving way, perhaps, to one of the important missing subjects mentioned above or even to differentiation inducers and give chemists something to really get their teeth into.

Although individual chapters are of very high quality and excellent reading for chemists, the overall balance and coverage of this book as the only representative of this series devoted to anticancer drugs leaves much to be desired. For the good chapters, however, it is a worthy addition to a medical chemist's library.

B.W. Fox